# Human Biological Monitoring of Diisononyl Phthalate and Diisodecyl Phthalate: A Review

**DOI:** 10.1155/2012/810501

**Published:** 2012-02-09

**Authors:** Gurusankar Saravanabhavan, Janine Murray

**Affiliations:** National Biomonitoring Section, Chemicals Surveillance Bureau, Health Canada, 269, Laurier Avenue, Ottawa, ON, Canada K1A 0K9

## Abstract

High molecular-weight phthalates, such as diisononyl phthalate (DINP), and diisodecyl phthalate (DIDP), are widely used as plasticizers in the manufacturing of polymers and consumer products. Human biological monitoring studies have employed the metabolites of DINP and DIDP as biomarkers to assess human exposure. In this review, we summarize and analyze publicly available scientific data on chemistry, metabolism, and excretion kinetics, of DINP and DIDP, to identify specific and sensitive metabolites. Human biological monitoring data on DINP and DIDP are scrutinised to assess the suitability of these metabolites as biomarkers of exposure. Results from studies carried out in animals and humans indicate that phthalates are metabolised rapidly and do not bioaccmulate. During Phase-I metabolism, ester hydrolysis of DINP and DIDP leads to the formation of hydrolytic monoesters. These primary metabolites undergo further oxidation reactions to produce secondary metabolites. Hence, the levels of secondary metabolites of DINP and DIDP in urine are found to be always higher than the primary metabolites. Results from human biological monitoring studies have shown that the secondary metabolites of DINP and DIDP in urine were detected in almost all tested samples, while the primary metabolites were detected in only about 10% of the samples. This indicates that the secondary metabolites are very sensitive biomarkers of DINP/DIDP exposure while primary metabolites are not. The NHANES data indicate that the median concentrations of MCIOP and MCINP (secondary metabolites of DINP and DIDP, resp.) at a population level are about 5.1 **μ**g/L and 2.7 **μ**g/L, respectively. Moreover, the available biological monitoring data suggest that infants/children are exposed to higher levels of phthalates than adults.

## 1. Introduction

Phthalates are a group of synthetic chemicals that are dialkyl or aryl/alkyl diesters of phthalic acid. Since the 1920s, phthalates have been widely used in the chemical industry in the manufacturing of polymers and a variety of consumer products. Currently, more than 12 billion lbs of phthalates are produced annually worldwide [1]. Highmolecular-weight phthalates, such as DINP and DIDP, have been mainly used as plasticizers in polymer manufacturing. Phthalate plasticizers are used to impart flexibility and durability to PVC polymers. Considerable amounts of DINP and DIDP are found in several industrial and consumer products such as construction materials, electrical wires and cables, automotive parts, clothing, and furniture [2–4]. As phthalates are not chemically bound to the polymers, there is a concern that they could leach out from the polymer matrix during usage [5]. Stringent government regulations in North America and Europe on the use of DEHP in consumer products have resulted in its substitution with other less toxic phthalates, notably DINP and DIDP [1]. This has prompted their inclusion in several national-level human biological monitoring surveys [6–10]. Earlier human biological monitoring studies have used MINP, the monoester metabolite of DINP, as a urinary biomarker to assess population exposure to DINP [7, 11, 12]. These studies have shown that the concentration of MINP in human urine is very low and often below the limit of detection of the analytical method employed. Researchers have speculated that such low concentration of MINP could be due to low human exposure to DINP or could indicate that MINP is not a sensitive biomarker for DINP exposure [7, 12]. Subsequent research on human metabolism and excretion of DINP has shown that most of the MINP formed is oxidized further to secondary metabolites prior to excretion [13, 14]. These results highlight the importance of choosing sensitive and specific biomarkers to assess human exposure to phthalates.

In this review, we summarize publicly available scientific data on chemistry, metabolism, and excretion kinetics, of DINP and DIDP. Major metabolites of DINP and DIDP that may be useful as exposure biomarkers in human biological monitoring studies are identified. Human biological monitoring data from around the world are analyzed to assess the suitability of specific metabolites of DINP and DIDP as exposure biomarkers for future biological monitoring studies.

## 2. Chemistry

Unlike DEHP, DINP and DIDP are mixtures of structurally similar chemical compounds. Two different compositions of DINP, namely, DINP-1 (CAS no. 68515-48-0) and DINP-2 (CAS no. 28553-12-0), are commercially available and are used interchangeably in industrial applications. Another DINP mixture, DINP-3 (CAS no. 28553-12-0), was previously in commerce but is now discontinued [15]. Like DINP, DIDP is also commercially available in two compositions (CAS nos. 68515-49-1 and 26761-40-0) which are fully interchangeable.

### 2.1. Production and Composition of DINP

Commercially available DINP is a complex mixture of diesters of *o*-phthalic acid containing C8–C10 (C9 rich) alkyl side chains. In the plasticizer industry, the term “iso” (as in di“iso”nonyl phthalate) refers to the “mixture of isomers” rather than the structural classification based on IUPAC nomenclature. DINP has an average molecular formula of C_26_H_42_O_4_ and molecular weight of 420.6 g/mol. Although DINP-1 and DINP-2 share these general characteristics, they are manufactured via two distinct chemical processes and hence their final chemical compositions differ considerably. DINP-1 is manufactured using the “polygas” process (as reported in [16]). In this process, propylene and butanes (n-butene and isobutene) undergo oligomerization to produce octene. Oxonation and hydrogenation of octene produces isononyl alcohols, mainly dimethyl heptanol-1, which is reacted with phthalic anhydride to obtain DINP-1. The DINP-2 manufacturing process utilises n-butene as the starting material. Here, n-butene is subjected to dimerization to yield isooctene. Oxonation and hydrogenation of isooctene primarily results in the formation of methyloctanols and dimethyl heptanols that are esterified with phthalic anhydride to yield DINP-2.

Exxon Mobil Chemicals and BASF are the major producers of DINP plasticizers in the world. Both chemical manufacturers have studied the purity and the main constituents of DINP. In terms of ester content, the purity of DINP-1 and DINP-2 are >99.5% [17]. However, the GC-MS analysis of pure DINP samples yielded multiple chromatographic peaks, indicating the presence of several structural isomers [18]. Koch et al. [19] analyzed isononyl alcohol (INA), used in the production of DINP-1 (INA-1) and DINP-2 (INA-2) using GC-MS, and found that INA-1 contains at least 15 isomers, while INA-2 contains >35 isomers. Moreover, they estimated that 4-methyloctanol-1 is the major isomer, constituting about 20% of INA-2 and 8.7% of INA-1. As INA-1 and INA-2 form the side chain of the final DINP-1 and DINP-2 mixtures, respectively, the above observations suggest that the 4-methyloctanol side chain dominates the DINP-1 and DINP-2 mixtures.

### 2.2. Production and Composition of DIDP

The two commercially available DIDP formulations are synthesised from the same starting materials, namely, propylene and butenes. The starting materials are subjected to oligomerization, followed by distillation to isolate C9 rich alkenes (nonenes). The alkenes undergo oxonation and hydrogenation processes to form linear and branched chain (C9–C11) alcohols, rich in C10 isomers. The alcohols undergo esterification with phthalic anhydride at high temperature (140°C–250°C) in the presence of a catalyst to form DIDP (as reported in [20]). Differences in the oxonation and esterification processes lead to the formation of two different DIDP formulations. However, for all practical purposes, these two formulations are completely interchangeable.

Like DINP, DIDP is a complex substance that consists of several structural isomers. DIDP has an average molecular weight of 446.68 g/mol and is represented by the molecular formula C_28_H_46_O_4_. Based on 1H-NMR analysis of isodecyl alcohol used in the manufacturing of DIDP, the commercial DIDP formulations are estimated to contain about 70–80% of dimethyl octanol side chains and the remaining 20–30% is made of methyl nonanols and trimethyl heptanols (<10%) ([21] as reported in [20]). The GC-MS analysis of the DIDP sample extracted from water showed the presence of at least 29 distinct chromatographic peaks. Rastogi [18] analyzed pure DIDP and DINP samples using GC-MS and found that both samples have multiple chromatographic peaks. Although DIDP chromatographic peaks eluted at slightly higher GC retention times, both DIDP and DINP showed overlapping chromatographic peaks suggesting the presence of common constituents.

## 3. Metabolism of DINP and DIDP

Due to the ubiquitous nature of phthalates, it has proven difficult to avoid external phthalate contamination during processing and analysis of environmental and biological samples. Moreover, as discussed below, phthalates are rapidly metabolized in humans. Therefore, in human biological monitoring studies, phthalate metabolites (as opposed to the parent phthalates) are measured as biomarkers of exposure [6, 9, 22]. Hence, an understanding of human metabolism and excretion kinetics of phthalates is crucial for identifying metabolites that are specific and sensitive to phthalate exposure. The urinary metabolic profiles of DINP/DIDP are complicated due to several factors. As stated earlier, the commercial DINP/DIDP formulation contains several structural isomers, and hence, exposure to such a mixture would result in the formation of many structurally similar metabolites. It is difficult to separate and identify individual isomeric metabolites, even with the state-of-the-art analytical techniques. To date, the identity of only some of these metabolites in human urine samples has been confirmed using high-performance liquid chromatography-tandem mass spectrometric technique [19]. In addition to this complexity, although DINP and DIDP predominantly contain compounds with C9 and C10 side chains, respectively, compounds with longer or shorter side chains are also detected in considerable amounts in their commercial formulations. For example, commercial DINP mixtures (DINP-1 and DINP-2) are known to contain DIDP (C10 side chain) and DNOP (C8 side chain) [16, 19]. This implies that exposure to DINP could produce DNOP and DIDP metabolites in addition to the metabolites that are specific to DINP.

**Figure 1:**
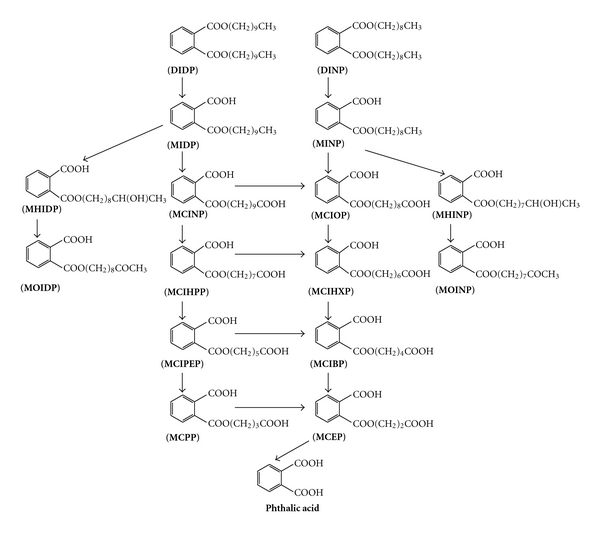
Proposed metabolic transformation of DINP and DIDP based on studies conducted in rodents and humans [6]. For simplicity, the linear side chains are depicted in phthalate structures in this figure. Legends: DIDP: diisodecyl phthalate; DINP: Diisononyl phthalate; MIDP: monoisodecyl phthalate; MINP: monoisononyl phthalate; MHIDP: monohydroxy isodecyl phthalate; MCINP: monocarboxy isononyl phthalate; MCIOP: monocarboxy isooctyl phthalate; MHINP: monohydroxy isononyl phthalate; MOIDP: monooxoisodecyl phthalate; MCIHPP: monocarboxy isoheptyl phthalate; MCIHXP: monocarboxy isohexyl phthalate; MOINP: monooxoisononyl phthalate; MCIPEP: monocarboxy isopentyl phthalate; MCIBP: monocarboxy isobutyl phthalate; MCPP: monocarboxy propyl phthalate; MCEP: monocarboxy ethyl phthalate [13, 14, 22, 23].

Studies on phthalate metabolism, conducted in rodents, as well as in humans, have shown that phthalates are metabolized very quickly and do not bioaccumulate [13, 14, 23, 24]. Despite differences in their physical and chemical properties, the metabolism of DINP/DIDP is very similar to that of DEHP [25, 26]. The proposed Phase-I metabolic pathway for DIDP and DINP in humans involves a preliminary ester hydrolysis step through which the hydrolytic monoesters (primary metabolites) are formed (Figure 1) [23]. The hydrolytic monoester undergoes oxidative metabolism via the *ω*-oxidation (oxidation at the terminal carbon atom of the side chain) or (*ω*-1) oxidation (oxidation at penultimate carbon atom of the side chain) pathway to form secondary metabolites with hydroxy-, oxo-, and carboxy-functional groups [19]. Metabolism of the secondary metabolites is possible and tertiary metabolites were also detected (Figure 1) [13, 14]. All these metabolites could also undergo Phase-II metabolism by conjugation with glucoronic acid and sulphonic acid to form respective conjugates before their elimination via urine.

Silva et al. [14] identified several primary and secondary metabolites of DINP in rat urine using HPLC(ESI)-MS/MS technique. The presence of MIDP, MNOP, and MCPP, in addition to MINP, was confirmed by comparing the chromatographic retention time and the mass fragmentation pattern of the metabolites with that of authentic standards. Using full-scan negative ion ESI mass spectra, they also identified several secondary metabolites of DINP: MCIOP (*ω*-oxidation product of DINP; Figure 1), as well as MHINP and MOINP ((*ω*-1) oxidation products; Figure 1). However, isomeric metabolites were not well resolved and their identification was based entirely on the mass spectral fragmentation patterns and specific MS/MS transitions. The authors also observed that the concentrations of secondary metabolites (such as MHINP and MOINP) of DINP were considerably higher than MINP. In addition to the DINP metabolites, secondary metabolites of DNOP (e.g., MCIHPP, MHIOP, MOIOP) and DIDP (e.g., MCINP, MHIDP) were also identified in this study based on specific MS/MS transitions. Thus, as stated in the previous paragraph, the interpretation of DINP metabolism is complicated by the presence of not only several structural isomers of DINP, but also by the presence of other phthalates such as DNOP and DIDP.

Kato et al. [13] followed the metabolism of commercial DIDP mixture in rats after oral administration of DIDP at a dose of 300 mg/kg-body. Urine samples were collected one day before dosing and three subsequent days immediately after dosing and analyzed for DIDP metabolites. The analytical method involved an enzymatic deconjugation step, followed by sample cleanup using online solid-phase extraction, and analysis using HPLC(ESI)-MS/MS technique. The secondary metabolites, such as MCINP and MHIDP, were the major metabolites (see Figure 1); the monoester metabolite, MIDP, was only present at trace levels. Besides DIDP metabolites, specific metabolites of DINP (e.g., MHINP) and DIUDP (e.g., MHIUDP) were also detected. The concentrations of the majority of metabolites in urine returned to baseline levels within 48 hours after dosing, suggesting a rapid clearance. Although the half-lives of individual metabolites were not reported, the authors suggest a half-life of about 14 h for all the metabolites.

Thus, studies on the metabolism of DINP and DIDP in rats suggest that these compounds are metabolised very rapidly. The primary metabolites of DINP/DIDP undergo extensive oxidation reactions during Phase-I to produce secondary metabolites. The DINP/DIDP urinary metabolic profiles suggest that the secondary metabolites dominate in urine while monoester metabolites (MINP and MIDP) are only minor metabolites. Metabolic and excretion studies carried out in humans (discussed in the next section) indicate that DINP/DIDP metabolic pathways in rats and humans are very similar.

## 4. Excretion of DINP and DIDP

Absorption, distribution, metabolism, and excretion studies conducted in rats show that after oral administration, the absorption of DINP/DIDP via gastrointestinal (GI) tract is very rapid, while their dermal absorption appears to be very minimal (<5%) [16, 24]. Once absorbed, DINP/DIDP predominantly distributes in blood, liver, and kidney. Initial ester hydrolysis of DINP/DIDP to their corresponding monoesters (MINP/MIDP) appears to happen in the GI tract; further oxidation of MINP/MIDP, to respective secondary metabolites (e.g., MHINP, and MOINP/MHIDP, and MOIDP), takes place in the liver [24]. The urinary metabolic profiles of DINP/DIDP are dominated by the secondary metabolites, and often no measurable concentrations of primary metabolites (MINP/MIDP) or the parent compounds (DINP/DINP) are detected [24]. On the other hand, in feces, most of the DINP/DIDP is excreted as parent compounds and a very small amount is excreted as secondary metabolites [16, 24].

Koch and Angerer [27] studied the urinary elimination kinetics of D4-ring labelled DINP-2 in humans by administering 98.2 mg of the substance orally (1.27 mg/kg-body weight) to an adult volunteer. The excretion of monoesters, as well as oxidized metabolites of D4-DINP, was analyzed in 22 urine samples collected during a span of 48 hours postdosing. The primary metabolites (MINP) and the secondary metabolites with hydroxy-, oxo-, and carboxy-functional groups were identified using respective D4-labelled standards. Elimination kinetics of D4-DINP in humans shows a biphasic pattern—a fast elimination phase (within 24 hr postdosing) followed by a relatively slow elimination phase after 24 hr. The elimination half-lives estimated for MINP and oxidized metabolites were 3 hr and 5 hr in the first phase, and 12 hr and 18 hr in the second phase, respectively. In the first elimination phase, the concentrations of hydroxy-metabolites were the highest, followed by the oxo-metabolites, and carboxy-metabolites. In the second elimination phase (>24 hr post dosing), the concentrations of carboxy-metabolites were higher than all other metabolites. The concentration of MINP was lower than the secondary metabolites at all sampling times. Moreover, almost all MINP was eliminated within 24 hr postdosing, while hydroxy- and oxo-metabolites were detected up to 48 hr postdosing. Carboxy-metabolites were detected even after 48 hr. The fractional excretion factor, defined as the molar ratio of the total amount of metabolite excreted to the applied dose of the parent compound, for MINP, MHINP, MOINP, and MCIOP were 0.02, 0.2, 0.1, and 0.1, respectively. These estimates also show that about 40% of the ingested DINP is excreted as hydroxy-, oxo-, and carboxy-metabolites within 48 h after dosing.

Recently, a similar study was carried out in 20 adult human volunteers (ten males and ten females) to assess the variability in DINP elimination kinetics due to differences in the ingested DINP dose and gender [23]. Under strict clinical control, these volunteers were administered two doses of D4-labelled DINP-2: 0.121 mg/kg body weight (high dose; 7.3 mg overall) and 0.012 mg/kg body weight (low dose; 0.78 mg overall). These doses are about ten to hundred times lower than the dose (1.27 mg/kg body weight; 98.2 mg) used in the previous study [27]. Urine samples were collected from the participants for up to 48 hr postdosing, at an interval of four hours between samples. All urine samples were analyzed for primary as well as secondary metabolites. More than 90% of each metabolite was excreted within 24 hours postdosing, suggesting rapid clearance. The concentrations of secondary metabolites (MHINP and MOINP) were higher than MINP in the urine samples collected during all time periods. The estimated elimination half-lives of the metabolites were between four and eight hours, which are slightly lower than the values reported in the previous study [27]. The fractional excretion factors for MINP, MHINP, MOINP, and MCIOP estimated in this study were 0.03, 0.12, 0.06, and 0.07, respectively. Two-way ANOVA analysis was performed to assess the effect of dose and gender on the excretion of DINP metabolites. The results show that gender did not have any statistically significant effect on the excretion of the DINP metabolites. The administered dose of DINP had a statistically significant effect on the excretion of MINP; however, such dose effects were not observed for other metabolites. Despite the fact that, in this study, the volunteers ingested about ten to hundred times lower dose of DINP than the single volunteer in the Koch and Angerer study, the overall metabolic profile and the excretion kinetics of the targeted metabolites in these two studies were very similar. 

The longer half-lives of secondary metabolites of DINP/DIDP together with their higher fractional excretion factors shown in the excretion studies indicate that the secondary metabolites are more sensitive biomarkers of phthalate exposure than the primary metabolites.

## 5. Biological Monitoring of DINP and DIDP

### 5.1. Exposure Biomarkers for Phthalates: General Considerations

Due to the widespread use of DIDP, DINP, and other phthalates in consumer products, humans are exposed to these compounds on a daily basis [5, 28, 29]. In the last decade, several studies have been carried out to assess the exposure of the general population to phthalates, to identify vulnerable populations, and to develop mitigation measures to reduce exposure [2, 13–16, 18, 20].

Human biological monitoring can be broadly defined as the measurement of a chemical, its metabolites, and/or its reaction products, in human body fluids and other tissues [30, 31]. The choice of biological matrix depends on several factors that include the study design, the characteristics of the chemical (e.g., physical and chemical properties, metabolism, excretion kinetics, etc.), and the availability of validated analytical methods. Traditionally, the concentrations of persistent pollutants (e.g., dioxins and furans) are measured in whole blood and blood components (such as plasma and serum), while nonpersistent pollutants (e.g., phthalates and environmental phenols) are measured in urine samples [30, 32]. Other biological matrices that have been considered for biological monitoring include saliva, breast milk, amniotic fluid, and seminal fluid [31, 33, 34].

Urine collection procedures are very simple, noninvasive, and straightforward. Moreover, large volumes of urine can be obtained with very little discomfort to the study participants. These attributes are desirable in large biological monitoring studies. However, as mentioned before, the detection of trace levels of phthalate diesters in urine samples is complicated due to its ubiquitous presence and rapid metabolism [35]. Hence, for biomonitoring purposes, phthalate metabolites are proposed as reliable biomarkers of exposure.

An ideal phthalate exposure biomarker would be a very stable metabolite with a relatively long half-life (*t*_1/2_) in the biological tissue/matrix (e.g., plasma, serum, urine) in which it is measured. The metabolite should also be stable during sampling, sample processing, and analysis. Furthermore, the metabolite should be specific to the parent phthalate of interest. Several high-molecular-weight phthalate diesters are known to have common metabolites (e.g., DINP, DNOP, and DIDP; Figure 1). Although analysis of such metabolites would be useful to establish phthalate exposure in general, detailed information on the metabolism and excretion kinetics of individual parent phthalates is required to assess their contribution to the measured concentrations of these metabolites. Finally, major metabolites of the parent phthalates would serve as good biomarkers, as their detection frequency would be higher.

### 5.2. Review of Human Biological Monitoring Data

The availability of biological monitoring data on DINP and DIDP is very limited. Concentrations of DINP and DIDP metabolites measured in biological monitoring studies conducted around the world are summarized in Table 1. Most of these studies were either carried out in a specific population (e.g., pregnant women, children) or with a relatively small sample size. Nevertheless, the metabolite concentrations reported in these studies are very similar. In general, the observed urinary concentrations of phthalate metabolites in biological monitoring studies are influenced by several factors, in addition to exposure. Some of these factors include the study design, age, time of sample collection (morning, afternoon and evening), nature of the samples (spot urine versus 24 h urine), and the analytical method used [30, 36, 37]. Hence, the data interpretation should be carried out with caution, as the observed differences in the concentrations of the metabolites may not necessarily reflect differences in exposure patterns.

Silva et al. [22] analyzed the concentrations of MINP, MHINP, MOINP, and MCIOP in 129 adult urine samples to assess the suitability of these metabolites as biomarkers for exposure assessments. MHIOP was detected in all the urine samples while MCIOP and MOINP were detected in 97% and 87% of the samples, respectively. On the other hand, the MINP concentration was below the detection limit (<0.36 ng/mL) in all the tested samples. They also noted that while MOINP was excreted in human urine predominantly as a glucuronide conjugate, MCIOP was excreted mostly as unconjugated (free form). The MHINP was excreted either as a conjugate or as a free compound. Recently, Calafat et al. [6] reported concentrations of MCIOP and MCINP in the United States general population (>6 years of age; 2005-2006 survey). These two metabolites were detected in about 90% of the tested samples at a median concentration of 5.10 ng/mL and 2.7 ng/mL, respectively. However, the monoester metabolite, MINP, was detected in only about 12% of the tested samples. In addition, the MINP concentrations in >80% of the samples analyzed during earlier U.S. national surveys (NHANES: 1999-2000, 2001-2002, 2003-2004) were below the limit of detection (0.8 *μ*g/L) [11]. In Canada, MINP was measured in urine of the general population (*n* = 3235) under the Canadian Health Measures Survey (CHMS; cycle-1; 2007–2009). Preliminary results from this survey suggest that MINP levels in >99% of the samples are below the analytical detection limit (0.4 *μ*g/L) [Saravanabhavan, unpublished data]. Even in the samples where MINP was found, the levels were very low (range: <LOD-3.34 ng/mL). This is consistent with other biological monitoring studies done around the world [22, 38–40]. Moreover, only very few biological monitoring studies have reported the concentrations of DIDP metabolites in human urine (Table 1). From studies in which both DINP and DIDP metabolites were measured, one could observe that the concentrations of secondary metabolites of DIDP were relatively lower compared to those of DINP metabolites [9, 32, 41]. The median urinary concentration of MCINP in the spot urine samples from a pregnant women cohort in Spain (2.8 *μ*g/L) [41] is very similar to the median concentration of MCINP (2.7 *μ*g/L) reported in the U.S. general population [41]. The median concentrations of MCINP from German studies (children: 1.3 *μ*g/L [9]; adults: 0.7 *μ*g/L [32]) were lower than the above data. Thus, the data from biological monitoring studies as well as the results from the metabolism and excretion studies discussed in the previous sections collectively suggest that MINP is not a sensitive exposure biomarker of DINP. A similar conclusion may be drawn about MIDP (monoester metabolite of DIDP), as both DINP and DIDP metabolism follow similar biochemical pathways. 

It is desirable to measure more than one secondary metabolite of DINP/DIDP in human biological monitoring studies to assess human exposure. However, due to logistic and economic reasons, it is not often feasible to measure multiple metabolites for each parent phthalate in large biological monitoring studies. The data in Table 1 suggest that, in general, the concentrations of secondary metabolites of DINP/DIDP are higher than the monoester metabolites. Among the secondary metabolites of DINP, the median concentrations of hydroxy-metabolites (MHINP), formed via the (*ω*-1) oxidation pathway, are the highest, followed by the carboxy- and oxo-metabolites (MCIOP and MOINP) (Table 1). In the case of DIDP, the concentrations of the carboxy-metabolites, formed via the *ω*-oxidation pathway, are the highest. Previous biological monitoring studies have shown that the concentrations of primary and secondary metabolites (e.g., MINP and MHINP) and the concentrations among the secondary metabolites (e.g., MHINP and MOINP; MHINP and MCIOP) in urine samples are highly correlated [6, 22], suggesting that these metabolites originated from the same parent compound (Figure 1). Hence, measurement of at least one secondary metabolite arising from the (*ω*-1) and *ω* oxidation pathways is recommended to assess human exposure to DINP/DIDP.

A few scientific studies have assessed the intra- and inter-individual variability in urinary metabolite concentrations. Fromme et al. [42] analyzed the concentrations of phthalate metabolites in 27 men and 23 women, aged between 14 and 60 years, living in Germany. To assess the daily variations, phthalate metabolites were measured in the morning urine samples collected from the participants over eight consecutive days. Several phthalate metabolites, including two secondary metabolites of DINP, namely, MHINP and MOINP, were tested. They observed a significant day-to-day variation in the metabolite concentrations. The interclass correlation coefficient (ICC), defined as the ratio of the inter-individual variance to the total variance, for MHINP and MOINP, were 0.31 and 0.33, respectively. Such low ICC values suggest the presence of considerable intra-individual variability in the metabolite concentrations. Hence, the authors suggest that the phthalate concentrations measured using single urine samples may not be adequate for exposure assessment.

For logistic reasons, national-level biological monitoring programs prefer collecting spot urine samples from study participants which are then analyzed for environmental chemicals including phthalate metabolites [6, 10, 43]. However, rapid clearance of DINP/DIDP metabolites in humans suggests that the metabolite concentrations in spot urine samples would reflect only recent exposure. Nevertheless, due to the analysis of a large number of samples in national-level biomonitoring studies, such as NHANES and CHMS, we speculate that the effects of iner-day and intra-day variations on the concentrations of phthalates in spot urine samples, measured at a population level, would be minimal.

Wittassek et al. [8] studied trends in the concentration of phthalate metabolites in young adults (mean age 24 years) residing in Germany by analysing biobanked urine samples collected from 1988 to 2003. They found that the concentrations of DINP metabolites (MHINP and MOINP) increased continually from 1988 to 2003, while the concentrations of DEHP metabolites decreased over the same time period. The authors suggest that this might reflect the fact that, in Europe, DEHP is being substituted by DINP in plastic manufacturing.

The results from NHANES (2005-2006) show that the urinary concentrations of MCIOP and MCINP have an inverse relationship with the age of the study participants; the concentrations of these metabolites were significantly higher in children compared to adults [6]. Similar results were reported from the GerES IV study conducted in Germany in which phthalate metabolites were analyzed in urine samples collected from 599 children, aged between 3 and 14 years [10]. The concentrations of secondary metabolites of DINP were significantly higher in toddlers (3–5 years) compared to children in higher age groups (6–8 years; 9–11 years; 12–14 years). Lin et al. [38] measured the concentrations of MHINP, MOINP, and MCIOP in spot urine samples from pregnant women, toddlers (2-3 years), and young children (5-6 years). The concentrations of all these metabolites were higher in the toddlers and children than in the pregnant women. Casas et al. [41] measured the concentrations of MCIOP and MCINP in spot urine samples from pregnant women and boys (4 years old) from five different cohorts. They found that the median concentration of these analytes were higher in the children than in the pregnant women. In this study, the boys and pregnant women cohorts were obtained from different geographical regions in Spain, thus the regional differences, in addition to age, may have contributed to the observed increase in concentrations of phthalate metabolites in boys. In a children's cohort study conducted in Germany, Koch et al. [9] measured the concentrations of secondary metabolites of DINP and DIDP, in addition to other phthalate metabolites, in urine samples from 111 children, aged between 5 and 6 years. In general, the concentrations of secondary metabolites of DINP/DIDP were lower than the secondary metabolites of DEHP. However, the estimated mean total daily intake values for DEHP, DINP, and DIDP were 4.5, 2.4, and 0.3 *μ*g/kg body weight/day, respectively, suggesting that the exposure to DINP/DIDP is significant and can increase the total phthalate body burden among children. All these biological monitoring data suggest that infants and young children are exposed to higher levels of DINP and DIDP than adults.

Previous exposure assessment studies have also suggested that infants could have high phthalate exposure [15, 16, 20]. PVC-based materials are commonly used in the manufacturing of toys designed for infants and young children. As phthalates are not chemically bound to PVC, the DINP/DIDP could migrate to the saliva during sucking and chewing of toys by infants, thereby increasing their exposure. The magnitude of DINP exposure in infants assessed by different researchers differs widely. This may be due to differences in the assumptions made on the use of toys, duration of mouthing, and the phthalate migration rates, risk assessment modeling, and so forth. As per European Union's risk assessment report, the estimated DINP exposure through the use of consumer products in infants (6 months to 3 years; assuming mouthing of toys) is higher (249.9 *μ*g/kg body weight/day; 95th percentile) than in adults (10.8 *μ*g/kg body weight/day; 95th percentile). In infants, mouthing of toys alone contributed to roughly 200 *μ*g/kg body weight/day of DINP exposure [16]. In 1998, Health Canada carried out a risk assessment on DINP in vinyl children's products [44]. Based on the scientific data available at that time, the DINP exposure in infants (3 months to 1 year) was estimated to range from 5 *μ*g/kg body weight/day to 458 *μ*g/kg body weight/day. However, the Dutch Consensus Group (RIVM) [45] estimated the DINP daily oral exposure of <26 *μ*g/kg body weight/day (95th percentile) in infants aged 3 months to 6 months through the use of teethers. The United States Consumer Products Safety Commission (CPSC) [46] has estimated DINP exposures in infants due to mouthing of toys as 94.3 *μ*g/kg body weight/day (3 to 12 months; 95th percentile) and 7.6 *μ*g/kg body weight/day (12 to 26 months; 95th percentile). In a recent reassessment, Babich et al. [47] estimated even lower DINP exposure levels in infants due to the use of PVC-based toys. Using the information on the migration rates and the mouthing duration from the CPSC observational study, the authors applied probabilistic methods to estimate DINP exposure. The daily DINP exposure in infants (at 95th percentile) was estimated to be 0.44 *μ*g/kg body weight/day (3 to 11 months), 0.53 *μ*g/kg body weight/day (12 to 23 months) and 0.56 *μ*g/kg body weight/day (24 to 36 months). In the case of DIDP, the European Union's risk assessment report estimated the exposure to be 226.5 *μ*g/kg body weight/day in infants (assuming mouthing of toys) of which the contribution from mouthing of toys alone is 200 *μ*g/kg body weight/day. The total exposure estimate of DIDP in children and adults (>3 years) is 5.8 *μ*g/kg body weight/day. Thus, both the biological monitoring studies and exposure assessments have identified infants and young children as vulnerable population subgroups for DINP/DIDP exposure.

## 6. Conclusion

DINP and DIDP are widely used as plasticizers in polymer manufacturing. Due to their relatively low toxicity, DINP and DIDP have been seen as suitable replacements for more toxic phthalates (such as DEHP) in the manufacturing of consumer products. As a result, there is an interest to assess the body burden of these phthalates occurring from nonoccupational exposure [22, 32]. Although the human biological monitoring data on DINP and DIDP are currently very limited, the available data suggest widespread human exposure to these compounds.

Several phthalate metabolites have been proposed as biomarkers to quantify phthalate exposure in biological monitoring studies. DINP, DIDP, and other high-molecular weight phthalates metabolize differently compared to low-molecular-weight phthalates, such as diethyl phthalate. The hydrolytic monoesters are the major metabolites of low-molecular-weight phthalates; however, the monoesters of high-molecular-weight phthalates undergo further oxidation to form secondary metabolites. Hence, monoester metabolites (MINP and MIDP) of DINP and DIDP are not detected frequently in human urine. The hydroxy- and keto-metabolites of DINP/DIDP are unique and can be formed only by the oxidation of respective monoester metabolites. Due to their high specificity, these metabolites are very useful in biological monitoring studies to assess human exposure to DINP/DIDP. On the other hand, the carboxy-metabolites, such as MCIOP, can be formed by the oxidation of more than one parent phthalate, suggesting poor specificity. However, one could speculate that the major proportion of any given carboxy-metabolite is derived from the monoester phthalate that is closer to it in the oxidation pathway. For example, the presence of MCIOP in human urine is a better indicator of exposure to DINP, although this compound may also be formed from DIDP. Overall, the secondary metabolites of DINP/DIDP are appropriate urinary biomarkers to assess human exposure to DINP and DIDP. The concentrations of secondary metabolites in infants and children are higher than in adults, which might indicate high exposure levels in infants/children. Moreover, the exposure estimates based on the concentrations of monoester metabolites of DINP and DIDP are likely to underestimate human exposure to these compounds.

## Figures and Tables

**Table 1:** Concentrations of DINP and DIDP metabolites measured in biological monitoring studies around the world. Legends: M: median; IQR: interquartile range; ND: nondetects; R: range; P95: 95th percentile; MINP: monoisononyl phthalate; MHINP: monohydroxy isononyl phthalate; MOINP: monooxoisononyl phthalate; MCIOP: monocarboxy isooctyl phthalate; MIDP: monoisodecyl phthalate; MHIDP: monohydroxy isodecyl phthalate; MOIDP: monooxoisodecyl phthalate; MCINP: monocarboxy isononyl phthalate.

Sampling year	Country	Population	Type of sample	*N*	DINP (ng/mL)				DIDP (ng/mL)				Reference
					MINP	MHiNP	MOiNP	MCiOP	MIDP	MHiDP	MOiDP	MCiNP	
2004/2008	Spain	Pregnant women (3rd trimester)	Spot urine	120	**	**	**	M = 4.0 IQR = 3.5 ND < 3%	**	**	**	M = 2.8 IQR = 2.2 ND < 3%	[38]
		Children (4 year old boys)	Spot urine	30	**	**	**	M = 7.5 IQR = 6.4 ND = 0%	**			M = 4.0 IQR = 4.1 ND = 0%	

2005/2006	Japan	Pregnant women (1st trimester)	Spot urine	50	<0.035	**	**	**	**	**	**	**	[35]

2001/2002 and 2006/2007	Taiwan	Pregnant women (3rd trimester)	Spot urine	100	**	M ≤ 0.25 R = 0.25− 364	M ≤ 0.25 R = 0.25− 288	M≤0.25 R = 0.25− 281	**	**	**	**	[33]
		Children (2-3 years)	Spot urine	30	**	M = 6.15 R = 0.25− 398.84	M = 3.84 R = 0.25− 287.46	M = 9.36 R = 0.25− 932.74	**	**	**	**	
		Children (5-6 years)	Spot urine	59	**	M = 7.94 R = 0.6− 1188	M = 4.3 R = 0.25− 352.62	M = 9.42 R = 1.22 − 915.6	**	**	**	**	

2007	Germany	Children (5-6 years)	Spot urine	111	**	M = 7 R≤0.25− 83.3 ND = 4%	M = 4.2 R≤0.25− 51.6 ND = 22%	M = 13.1 R≤0.25 −168 ND = 1%	**	M = 0.4 R≤0.2−9.8 ND=40%	M≤0.25 R≤0.25− 3.4 ND = 70%	M = 1.3 R ≤0.25 −16.0 ND = 6%	[12]

2003/2006	Germany	Children (3–14 years)	Morning urine	599	**	M = 11.0 R≥0.25− 198 ND = 0%	M = 5.4 R≥0.25− 86.7 ND = 2%	M = 12.7 R ≥0.25− 195 ND = 0%	**	**	**	**	[13]

2006/2008	Denmark	Children (6–21 years)	24 hour urine	129	Sum of all metabolites: M = 31 R = 3− 281 ND = 0%				**	**	**	**	[34]

1988/2003	Germany	Students (21–29 years; 326 females and 308 males)	24 hour urine	634	**	M = 11.9 R ≤ 0.25− 85.4 ND = 1%	M = 1.0 R ≤ 0.25− 63.8 ND = 8%	**	**	**	**	**	[11]

2005	USA	Adults	Spot urine	129	<0.36	M = 13.2 R = 1.4 −202.7 ND = 0%	M = 1.2 R≤ 0.25− 201.7 ND = 13%	M = 8.4 R≤ 0.25− 310.8 ND = 3%	**	**	**	**	[27]

2007	Germany	Adults	Morning urine	45	**	M = 4.7 P95 = 16.8	M = 1.7 P95 = 6.7	M = 5.3 P95 = 15.5	**	M = 1.0 P95 = 4.0	M = 0.2 P95 = 1.1	M = 0.7 P95 = 2.6	[39]

1988/1994	USA	Adults (20–60 years)	Spot urine	289	M ≤ 0.8 R ≤ 0.8 −79.7 ND ≥75%	**	**	**	**	**	**	**	[10]

2005	Germany	Males (14–60)	Morning urine (men)	23	**	M = 5.5 R = 2.2−49.4 ND = 1%	M = 3.0 R=0.5−8.2 ND = 2%						[40]
		Females (14–60)	Morning urine (women)	27	**	M = 5.7 R = 0.7−12.1 ND = 1%	M = 3.1 R = 3.8−23.6 ND = 2%	**	**	**	**	**	

Unknown	Germany	general population (6–80 years)	Spot urine	102	**	M = 2.0	M = 1.3	M = 4.0	**	**	**	**	Koch et al. unpublished data

2005/2006	USA	general population (≥6 years)	Spot urine	2548	<0.8	**	**	M = 5.10 R = 0.7− 4961 ND = 5%	**	**	**	M = 2.7 R ≥0.6− 672.6 ND = 10%	[9]

2007–2009	Canada	general population (6–59 years)	Spot urine		<0.4	**	**	**	**	**	**	**	Saravanabhavan, unpublished data

## Acronyms and Abbreviations

CHMS: Canadian Health Measures Survey
DEHP: Di-(2-ethylhexyl) phthalate
DNOP: Di-n-octyl phthalate
DINP: Diisononyl phthalate
DIDP: Diisodecyl phthalate
MNOP: Mono-n-octyl phthalate
MHIOP: Monohydroxy isooctyl phthalate
MOIOP: Monooxoisooctyl phthalate
MCIHPP: Monocarboxy isoheptyl phthalate
MCPP: Monocarboxy propyl phthalate
MINP: Monoisononyl phthalate
MHINP: Monohydroxy isononyl phthalate
MOINP: Monooxoisononyl phthalate
MCIOP: Monocarboxy isooctyl phthalate
MIDP: Monoisodecyl phthalate
MHIDP: Monohydroxy isodecyl phthalate
MOIDP: Monooxoisodecyl phthalate
MCINP: Monocarboxy isononyl phthalate
MHIUDP: Monohydroxy isoundecyl phthalate
LOD: Limit of detection
NHANES: National Health and Nutrition Examination Survey
GerES: German Environmental Survey
PVC: Polyvinyl chloride
GC-MS: Gas chromatography-mass spectrometry
HPLC: High-performance liquid chromatography
NMR: Nuclear magnetic resonance spectroscopy
ESI: Electrospray ionisation.
